# “I used to be as fit as a linnet” – Beliefs, attitudes, and environmental supportiveness for physical activity in former mining areas in the North-East of England^☆^

**DOI:** 10.1016/j.socscimed.2014.12.002

**Published:** 2015-02

**Authors:** Esther Rind, Andy Jones

**Affiliations:** a School of Environmental Sciences, University of East Anglia, Norwich NR4 7TJ, UK; b Norwich Medical School, University of East Anglia, Norwich NR4 7TJ, UK

**Keywords:** England, Physical activity, Industrial decline, Former mining communities, Changing social and physical environments, Focus groups

## Abstract

Studies of geographical variations in physical activity behaviours have suggested that activity levels are particularly low in areas that have undergone employment loss associated with the decline of industry. This is of concern given that affected populations are already at risk of poor health. Applying focus group methodology amongst 19 participants in four groups, this study aims to unpack how broader societal and environmental changes associated with industrial decline affect beliefs and attitudes towards physical activity in ex-mining communities in the North-East of England. Identified core themes comprise the direct impact of deindustrialisation on social and physical environments. Based on our findings, we provide evidence for mechanisms that operate via loss of occupational physical activity as well as the progressive development of environments that are not fit to support population activity levels. Particularly important was the loss of recreational facilities, public green spaces and sports facilities that were owned and organised by the miners themselves with support from the mining companies. Attitudes and beliefs directly related to the areas' industrial past were also seen to be key. We suggest that the development of interventions considering the socio-cultural history and socio-economic reality of communities could be a promising route to encourage more active lifestyles in deprived areas with particularly low levels of physical activity.

## Highlights

• Reasons for less physical activity previously observed in ex-mining areas unclear.
• Detailed focus group interviews with community residents to explore influences.
• Cultures of being physically fit and active present in the past have been lost.
• Loss of recreational facilities and opportunities associated with colliery closure.
• Important to consider socio-cultural histories in physical activity interventions.

## 1 Introduction

In recent decades, levels of physical activity (PA) have declined considerably throughout many European countries (World Health Organisation, 2006). In England, activity participation is particularly low, and in 2012 60% of the population did not achieve the recommended levels of PA, a figure that has not changed substantially since 2008 (Craig et al., 2009; Health and Social Care Information Centre, 2013). The reasons behind PA declines are varied but there is evidence that they may be partly associated with the broader social and economic effects of industrial decline and restructuring, which includes the transition from a heavy labour-based industry to a service-based and information-orientated society (Bazen and Thirlwall, 1997). For example, previous work has illustrated that the populations of English districts that have undergone a particular strong transition from industrial to post-industrial economies are more likely to report low levels of PA than those which have not seen such changes (Blaxter, 1990, Ellis et al., 2007; Rind et al., 2014). Indeed the authors have previously described substantial geographical variations in recreational PA in England (Rind and Jones, 2011).

**Fig. 1:**
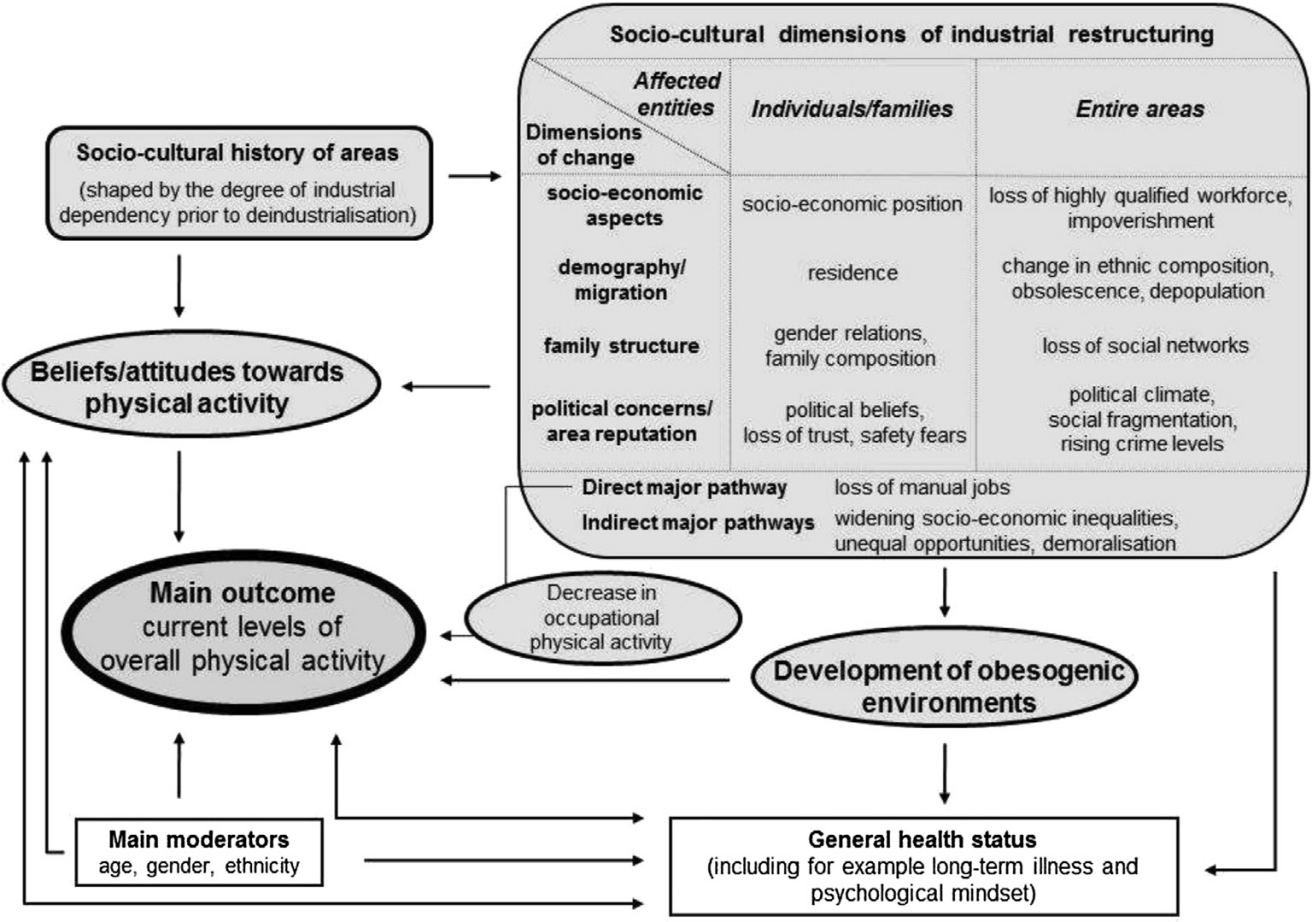
A conceptual framework linking PA to socio-cultural dimensions of industrial decline.

Whilst there is good evidence that the populations of previously industrialised areas show lower levels of activity, the particular causal mechanisms linking socio-cultural characteristics of the post-industrial society to PA are, however, not well understood. Based on ecosocial theory (Krieger, 2001) which considers how present and historical physical, social and cultural conditions impact population patterns of health, we recently developed a conceptual framework linking PA to socio-cultural dimensions of industrial decline (Rind and Jones, 2014) (see Fig. 1). It considers the decline of labour-intensive jobs as a direct pathway which, if not counterbalanced by an increase of PA within other activity domains, will lead to overall activity declines in those areas affected. The effects of losses of work related PA may be particularly strongly felt if areas of industrial decline hold inherited cultures and regional identities that are resistant to change and the adoption of alternative PA behaviours. Indeed, in the late 1940s Ferdynand Zweig (1948) published a detailed portrayal of English pitmen, writing that “*The force of habit is nowhere stronger than in the colliery and the mining village. A change of habits is most difficult to accomplish, even if the change proves beneficial and the habit has no longer a functional value.*” (Zweig, 1948: 8). This raises the question as to the capacity of these communities to adapt to the very substantial changes to the social and physical environment that have been associated with the loss of such industries.

Perhaps more importantly from a population perspective, the framework incorporates ways through which the socio-cultural history of areas may shape beliefs and attitudes towards PA, and how the changes observed in local environments may reduce opportunities for a sufficiently active lifestyle. For example, activity behaviours may be influenced through role models established by peer, friend, family and community networks which are, in turn, related to socio-cultural backgrounds (Legh-Jones and Moore, 2012; Yu et al., 2011). This may be problematic if industrial decline and increasing socio-economic inequalities act to weaken established community and family structures via high unemployment, demoralisation, obsolescence and movement of labour (Altena and van der Linden, 2002).

There may also be influences associated with physical decline due to a prior history of manual labour. Living in an area with a history of mining for example has generally been associated with a relatively high risk of poor health (Shucksmith et al., 2010), which is, in turn, likely to directly impact levels of PA. For example, for some time it has been recognised that overall mortality rates in former coalfield areas were higher than those for Great Britain generally, and that levels of reported limiting long term illness are greater than would be expected based on the population characteristics of such localities (Fitzpatrick et al., 2000; Wiggins et al., 1998). More recently, Riva et al. (2011) used data from the 2004–2006 Health Surveys for England to examine whether poorer health outcomes still characterise coalfield areas today. They found that those residing in these areas were significantly more likely to report limiting long term illness and less than good health although there was substantial variability within communities, suggesting local conditions were important. The potential impact of these health disparities on physical activity in these communities is not known.

If activity declines are to be reversed at a population level, we suggest an important first step is to identify likely causal mechanisms that operate on the ground. A number of recent studies have suggested individual (e.g. lack of time and money, cultural background, health problems, social isolation, safety fears) as well as contextual (e.g. neighbourhood support, community participation, physical environment) factors as important barriers to being active (Allender et al., 2006; Hoebeke, 2008; e.g. McNeill et al., 2006; Wendel-Vos et al., 2007). It may be that some of these factors are acting to mediate the association between the losses of manual employment and declining physical activity, but it is unknown which may be important and to what degree.

Using a qualitative approach, this study explores the implications of industrial decline on the opportunities for physical activity and attitudes towards activity of the residents of effected areas. We focus on coal mining communities, once at the heart of the UK economy but experiencing harsh economic decline since the mid-1970s due to pit closures. The 170,000 jobs lost between 1984 and 1997 represent a quarter of all male employment in affected areas (Riva et al., 2011). By undertaking focus groups with 19 residents of ex-mining communities in the North-East region of England, we aim to gather a better understanding of how the process of employment decline in the physically demanding mining industry may have led to structural, socio-cultural and environmental changes in former pit communities, and whether this may have contributed to the generation of cultures of non-participation in PA. We argue that gaining such understanding by talking to those who have direct experience of the process and consequences of industrial decline will inform theoretical models that underpin work relating cultures to health behaviours and contribute to a better understanding of the specific mechanisms underlying spatial variations in PA, thus aiding the development of effective PA interventions.

## 2 Methods

### 2.1 Research design

This research focuses on socio-cultural and environmental changes in former mining communities and how this has impacted community life as well as health-related behaviours, including PA. We used a case study approach that applied focus group methodologies to permit the exploration of narrative accounts of individual lives and experiences (Elliott, 2005) and to understand how individuals view changes in their community relative to others. Focus groups were particularly chosen instead of 1:1 interviews as a number of the issues we were interested in examining were associated with the collective norms and beliefs of communities and the discussions that can take place in focus groups can help bring out such themes (Kitzinger, 1995). Furthermore, focus groups provide a good insight into how relationships between people and places affect practices of everyday life (Kitzinger, 1994; Rabiee, 2004), and Khan and Manderson explicitly highlight their value in understanding dimensions of health and illness in village settings (Khan and Manderson, 1992). This study was approved by the Research Ethics Committee of the University of East Anglia.

### 2.2 Study area

This study took place in the North-East of England across the Durham and Northumberland Coalfields, which have a significant history of employment decline in the mining sector. In terms of several indicators of deprivation, including employment, education and health, the region performs poorly compared with national trends (O'Donnell et al., 2008; Walsh et al., 2008). Within the region, two case study communities were selected; one was a semi-rural area within the Derwentside District (population 89,191) and the other a semi-urban area within the Sunderland District (population 283,509) (Office for National Statistics, 2010). Both localities have a history of coal mining, but since the 1980s all mining-related activity ceased, and the sector no longer provides local employment (Beatty et al., 2005). In terms of PA, previous analysis showed that both areas have relatively low levels of recreational PA and walking, as well as a high prevalence of non-active residents (Rind and Jones, 2011).

### 2.3 Selection of participants and procedure

The discussions were conducted with participants who had lived in the area for at least 30 years to ensure that they were able to provide information on potential environmental, socio-economic and health-related changes since the 1980s. Further, this allowed the discussions to take place within familiar social networks and environments where participants spontaneously explored and discussed issues and concerns related to changes in community life. In order to aid participant recruitment, we offered to pay each focus group attendee £25 to cover their time and travel expenses. All participants were recruited via flyers from a local working men's club in each study location as well as word of mouth. Working men's clubs are a type of private social club first created in the 19th century in industrial areas of the United Kingdom, particularly the North of England, the Midlands and many parts of the South Wales valleys, to provide recreation and education for working class men and their families. They are hence historically rooted in the areas' industrial past (Bulmer, 1978), being owned by their members, and provide education, social entertainment and opportunities for recreation. Initial contact with the clubs was made through the Durham Branch of the Working Men's Club and Institute Union, which is a voluntary association representing and supporting affiliated clubs and its members (WMCIU, 2011). Recruitment continued until the target sample size was reached.

During August 2011, four 1–1.5 h group discussions with a total of 19 participants were conducted. The discussions were organised separately for men and women to specifically explore male and female attitudes and beliefs towards community life and health-related behaviours. All sessions took place in the clubs. Before the closure of each session, participants completed a short questionnaire (additional material A) providing socio-demographic and health-related background. Furthermore, there was the opportunity to explore the areas' industrial past over a two week fieldwork period that included informal discussions with local residents and members of several other working men's clubs. Field notes taken over the period of study were used to assist with interpretation of the information gained from the focus groups.

### 2.4 Questioning route

To drive the discussion, a questioning route (additional material B) was developed following the methodology of Krueger and Casey (2000). The introductory theme explored general aspects of community life (e.g. “*In what sort of things do you get involved in your community?*”). The transition theme covered residents' perceptions and opinions on health differences between places generally and specifically within their communities (e.g. “*In some places people have worse health than in others. Can you think of any reason for that?*”). This provided insight about local health issues and conditions that may have changed over time.

The key theme targeted perceptions and opinions of participants on health behaviours in their community, with a focus on PA. In order to initiate and facilitate this discussion, the participants were asked to choose from a range of 20 newspaper headlines to act as stimulus material, which had an even number of positive as well as negative connotations related to health and PA (e.g. positive: “*Gardening for health*”, negative: “*Local park needs makeover*”), and they were asked to present their thoughts. Other authors, such as Liamputtong (2012), recommend this technique, as the process requires the participants to establish individual priorities and provided insight into whether these were shared throughout the group. The closing theme summarised the discussion and provided an opportunity for the participants to add yet undiscussed areas of concern.

### 2.5 Data management and analysis

The focus groups were recorded and transcribed verbatim using f4, version 4.2 (Dresing and Pehl GmbH, 2010). To protect the participants' confidentiality, we used pseudonyms and omitted local place names. The transcripts were analysed following Krueger's (2000) framework of data analysis, incorporating key stages of framework analysis by Ritchie and Spencer (1994). This approach follows a continuous and overlapping analysis sequence including familiarisation with the data, identifying a thematic framework, managing and coding the data, and linking individual quotes to put identified core issues into context (Krueger and Casey, 2000; Rabiee, 2004; Ritchie and Spencer, 1994).

## 3 Results

Ten men (M) and nine women (W) attended the focus group (FG1–FG4) discussions. Characteristics of the study sample are summarised in Table 1.

The average age of the participants was 72 years, and all were of a white ethnic background. All but two were retired, and nine out of the ten men had a working history related to the mining industry. A number of the female participants were married to former miners. Most participants characterised their health status as “fair”, with only women reporting “good health”. Only residents of the more urban area characterised their health status as “bad”.

We identified and subsequently present two main themes that evolved from the focus group discussions. Firstly, we report the participants' opinions on how industrial change directly impacted different PA domains, including those of occupational, domestic, and leisure time activities. Subsequently, we present how participants discussed activity-related changes in social and physical environments, including impacts on family and community life, as well as on the physical environment.

### 3.1 Direct impact of industrial decline on different activity domains

#### 3.1.1 Occupational and domestic activities

All participants discussed the mining industry's physically demanding working environment, summarised by one of the male attendees who also discussed broader contexts of industrial restructuring such as the development of a service and information orientated society (FG2):

M2: [...] you can never explain to people, what you might call modern day things like school teachers and work places that make little telephones and things like that, you know, with the greatest respect, trying to tell these people what being in the mining industry or heavy engineering, what it meant on a day to day basis. And some of the things you had to do, just to get the job done, [...], to produce coal, [...], just to produce, it was so hard, the work, and trying to explain that to people.

For most of the male participants, working life started at an early age, there was not much choice and several stayed in manual occupations until retirement (FG3):

M3: There was the colliery [mine]. Coal, that was it.

M1: I left school on a Friday, and my father said you are coming to the pit [mine] on the Saturday. [...]. 15 years old, and I started the pit, on a Monday. [...]. That was where you [were] born into.

For whole regions, the demise of the industry resulted in a direct loss of occupational PA. Although some of the miners found work in alternative industries such as the shipyards, factories or building work, most of these jobs had gone by the end of the 1980s (FG1). Changing jobs and adjusting to new working environments also had psychological effects through increased stress levels resulting from losing the security of familiar working environments (FG2):

M3: The difference in working down the pits when you say the word “marrer” [work mate], you were marrers because you had to depend on each other. But when you are not in the pits, but in factories they stab you in the back the whole weekend. It wasn't like that down the pits. [...].

M1: But it was very difficult for the lads. I mean, they were paid off, and made redundant and things like that. [...].

M3: Your whole lifestyle changed, didn't it?

M1: Some of the lads went into stress mode because they were doing jobs that were out of their kind of style. [...].

M2: Invariably people went on what you might call heavy engineering into a lighter type of facility. [...]. [T]here was no way you could go into what you might call a new industry and expect to work as hard as you were because somebody would stop you and say, hang on, this is the way we do it here. [...] and a lot of the places you were going to, you weren't allowed to lift anything. That was a crime to do that, you know. So, really, it wasn't something you control, you were controlled by the system that you fit into, you know. [...].

M2: The men were different. And the more, the more collieries issued redundancy, the more you moved on, the more you moved on. [...].

M1: They were industrial gypsies, that's what they were, they just moved from one pit to another.

Today, most of the participants report suffering from long-term illnesses associated with heavy manual work and a dangerous working and living environment (FG2). A female participant clearly linked particular diseases to local pollution (FG1):

F3: Yes, [around here] there are lots of people who've got asbestosis, silicosis, all the, how can I say it, all the diseases that's not curable. They've all got that, where anywhere down London or Devon or places like that they haven't got it. Because they're not breathing the dust air. That's round about here you know.

Another female participant who worked in the local hospital summarised (FG4):

F2: Well, a lot of mining injuries it was, mainly, you know, a lot of you know broken bones and falls and dust and things like that. Heavy work, these men have worked hard. [...]. Their knees, with crawling in the low, that was a lot of the injuries that they came in with. Mining injuries and their backs. And I think that is where most of the men suffer now. And then their chest with the dust.

The women also emphasised that many of their relatives who worked in the collieries died before or shortly after retirement (FG1).

Although none of the female participants were actively involved in occupations related to the mining industry, most of them used to work in environments that required them to be physically active (e.g. factory, commercial kitchen, health care facility, market trade). Furthermore, all of the women have been responsible for most of the domestic activities such as baking, shopping, washing or ironing (FG1). In the past, these activities required more physical strength than nowadays (FG4):

F2: [...] I was strong as a horse when I was young [...]. And then we used to have a big ton of coal, the coalman was coming and empty the coal outside your gate.

F1: You had to fill those buckets.

F2: [...]. You did things like that. You wouldn't dream doing anything like that nowadays, would you? God, no no. You have got any amenity now, you see, and an easier life.

However, they also discussed how, in particular for younger residents, changing lifestyles may act as a barrier to continue long-established activities such as having an allotment (FG4):

F3: [Hannah] had an allotment [vegetable garden] down the road. And then [her partner] went to [...] work [further away]. And she sets it on for years, but then it got too much for her. Too much for her, working and then having the three kids to look after and the house and that. So she packed it in. [...].

#### 3.1.2 Leisure, recreation and sport

Although most of the participants described their working life as physically challenging, they also talked about being relatively active during leisure time. Coping with the hard working environment required physical fitness (FG1):

F2: My dad had to go on a diet because he was big and the seam [mine tunnel] was that low, he couldn't get in [...].

Moderator: So they were all pretty fit when they used to work in the pit?

F2: Oh yes. You had to be. It was hard work.

M3: I used to walk the greyhounds. I used to walk 25 hours a day before I come over here. Because you had to train them, right. And I tell you I was as fit as a linnet [type of bird often kept by miners].

The colliery environment was directly related to the provision and maintenance of recreational opportunities (FG2):

M1: [...]. [Around here] the provision for football pitches, cricket pitches, bowling greens, meeting places, and all that was provided by the miners by the reduction of their pay code. The city or the district councils they provided nothing in terms of leisure facilities or anything, they didn't, nothing. [...].

The closure of the pits had therefore a direct effect on opportunities for leisure and recreation (FG3):

M3: Oh, Tuesdays used to be enormous. That's the biggest loss in this village. We had a tennis court. We had a bowling green. We had two football teams and we weren't any down [short of players].

M4: There is no sport and activity since the colliery ceased. [...].

Several of the participants repeatedly highlighted the importance of walking for pleasure and gardening in their allotments. Indeed, there was discussion suggesting that not enough provision was available. For example.

M2: It's a big part. You can't get an allotment anywhere. You couldn't get an allotment now. That doesn't mean that there is enough allotments. There is a lot of people who would like an allotment and haven't got one.

However, for most of the participants, long-term illness was a severe impairment to maintain an active lifestyle throughout retirement. Although mining activity-related health impairments particularly concern men, many women have been affected by dust exposure and environmental pollution.

The participants also worried about difficulties to motivate new and younger residents to participate in voluntary community work. Most participants regarded their local Working Men's Club as the focal facility within their community as here many of the social activities were organised and also took place. However, with the closure of the pits and declining memberships many clubs had to close or struggle to remain open which resulted in a loss of sociability for the affected communities. The participants also discussed tedious bureaucratic processes and costly construction works as constraints to improve the local park environment. Another important issue was the lack of recreational opportunities for the younger generation (FG3):

M2: Sport and recreation that is the main thing. There is nothing here for the kids.

M3: We have the football field, but they didn't play.

M4: You can get these things but you have got to get someone to organise them. Someone who'll be dedicated to the job. It's hard to come by to get some people [...], in days gone by they were there, you could pull them off a tree, but now, you have just got to find them. And if you are involved with young children and the youth, the police, you know, you have to have a certificate to see you can do this. It's very, very difficult. ‘Cause, you know, if something goes wrong when you are dealing with the younger generation, it's very bad trouble on. Very bad, you know.

M2: [...]. And there is health and safety.

M3: That's a killer.

### 3.2 Changes in social and physical environments

#### 3.2.1 Family and community life

All focus groups discussed the loss of social networks and how this altered community and family life over the past decades. With the progressive closure of the pits, several of the old-established families were forced to take up work in other areas (FG3):

M1: [...]. People went to Yorkshire, Nottingham and Wales. The miners, you know. I had five cousins, have never seen [them] since 1962.

M2: Years ago it was all miners that lived here, wasn't it, mostly. I would say 90% worked down the pit [...].

The collieries played a pivotal role, not only in providing employment, but also in shaping close–knit relationships between the pit families. The decline of the industry and continuous migration dissolved consolidated socio-economic structures (FG2):

M2: So, it's not as if, in the olden days you would be talking about a family arrangement, collieries and colliery villages you might call a family village, or a family pit, this kind of thing. It doesn't happen now because there has been an influx of so many people from so many places [...]. So, to me it's completely changed from the days when it was what you might call a family pit village type scenario. Completely changed.

Even though several of the participants were relatively happy with their current neighbourhood settings, they emphasised how the situation has changed compared to the times when the mines were still running (FG2):

M1: No, the mix [of people] is quite well. Not as well as they did when the mines were going. You live together, you eat together, you stay together, you work together, you know and that was it.

Another group elaborated the issue of increasing anonymity (FG1):

F3: Well, in the olden days the community was community people; they loved one another. Now, where I am living, I am ok, but some people don't even know the next door neighbours, you know, that is changed, because you're frightened for who you're gonna get next door. Whether you're going to get a nice quiet family or a rowdy family or what. For when we were little, we were excited what we were going to get, ‘cause we were all sitting in the same boat.

“Sitting in the same boat” was later referred to as experiencing economic equality (FG1):

F2: Everybody was poor. Everybody was the same. But now there are people that are poor and people that have a lot of money, you know. It separates.

Increasing socio-economic inequalities were directly related to changes in family life, and the in one focus group participants described families outside their village being increasingly confronted with unstable economic as well as social circumstances (FG4). All groups were particularly concerned about the loss of community spirit (FG2):

M6: There is a lot of them [local residents] that don't do self and look after number one. I think that was preached by one of our old esteemed prime ministers, wasn't it?

M3: Maggie Thatcher [ex-British prime-minister associated with the closure of many mines in the 1980s]. [Expletive deleted.]

M6: Maggie Thatcher, look after number one. And a lot of it, a lot of it is still what we [do], we just look after themselves and that's it.

#### 3.2.2 The physical environment and access to health care facilities

The closure of the pits had effects on the physical environment in both study areas. All participants discussed the disappearance of coal heaps and the reduction of air pollution which they feel impacted positively on health. They also discussed how the improvement of health services may relate to the health of residents (FG3):

M3: I think the generation coming is a lot healthier than what we are.

M2: Well, I think when you look around and keep notes in general, see people from our age and [younger people] going by, those are quite a big lad, and us three, we are on a smaller statue. Seeing the young lads, they are all bloody giants.

Moderator: And why do you think is that?

M3: Well, because they haven't got the dust, [...], the heaps is gone, everything is gone.

M2: Well, and the NHS [National Health Service] is one of the more brilliant things. Without the NHS the best part of us wouldn't have been here [...].

M1: Yes, I wouldn't be sitting here.

Participants further discussed that the “greener environment”, including the decommissioning of an old railway line, has resulted in increased participation of cycling and walking throughout the area, which was seen as being particularly important for younger families.

Another participant summarised changes which impact mobility within local communities (FG2):

M2: [...]. The speed of things changed. The facilities to account for that also change. You couldn't have narrow little roads to get from A to B, like you used to. A, the transport system wasn't required [...]. You didn't travel very far, you know. Everything evolved around where you were at. The village you were in and the next neighbour village that was a night out. [...], technology has speeded everybody up, [...], you have got motor race, you have got wide roads, you have got improved traffic, cars are better, buses are better, more reliability, more numbers of them, you know. [...].

## 4 Discussion

Earlier studies have investigated socio-cultural change in mining areas from a primarily social perspective (Bulmer, 1978; Waddington et al., 2001), and have focused on health inequalities (Riva et al., 2011; Shucksmith et al., 2010) or have emphasised the importance of social capital in relation to health (Gilbertson et al., 2005; Gilbertson and Manning, 2006). To our knowledge, the present study is the first attempting to link several dimensions known to impact PA within a particular historical context and defined community setting. This provides an in-depth picture of changes in activity-related environments in ex-mining communities where adverse socio-economic and other legacies persist to create what may be worse ramifications compared to other areas with an industrial past.

The results of our study provide insight into the mechanisms that may be present in former mining communities may underlie the previously observed lower levels of physical activity in these populations. In doing so we sought to add evidence to a previously published theoretical framework. Many of the issues discussed crossed rigid categories which highlighted the interrelationship between multiple dimensions of the areas' industrial past and PA.

Our framework had suggested that the physically demanding nature of employment in industry might have led to cultures of non-participation in physical activity which persist today. However, from our focus groups, we found rather little evidence to suggest this process was operating. The men and women we spoke to discussed the physically demanding nature of the work, both in the pits and in the home, that they were expected to do in the past. However participants, and in particular the men, spoke about how physically active they were outside work when the collieries were in operation and how the physical fitness instilled in them from their work benefitted them during their recreational activities.

Our conceptual framework had posited that poor environmental quality in ex-mining areas might create neighbourhoods were being physically active was not an easy option. However, there was rather limited evidence that our participants agreed. Although the decline of recreational opportunities was discussed, there was a general view that regeneration initiatives in these communities did have a measureable effect of neighbourhoods. Nevertheless, they did not unanimously associate this with increased opportunities for activities such as recreational walking, because long-term illness and safety fears regarding traffic and crime limited their activity potential and motivation in the first place. Our results also showed that issues related to health and safety (such as the need for a criminal records bureau check), maintenance (difficulties in recruitment of volunteers), and bureaucratic barriers (lack of monetary support) were seen as important factors influencing PA opportunities, partly also affecting children and adolescents.

In particular, the results of this study emphasise how the complete disappearance of the mining industry has left a health legacy primarily affecting the activity potential of the older generation. A theme throughout all discussions was the loss of trust and social networks in both of the communities, which has previously been shown to be an important determinant of physical activity (Legh-Jones and Moore, 2012; Lindström, 2011; McNeill et al., 2006). Several participants recalled former community and family life according to the sociological models of the mining community (Bulmer, 1975) which describe work, family life and recreation in the traditional pit communities as closely intertwined. In terms of social structure, working and living environments in mining communities were largely homogeneous and collectively organised. All of the families shared the same experiences, gender roles were clearly defined and everyday life and leisure time were organised around the colliery. This promoted social cohesion and mitigated against the exceptionally hard working and living conditions affecting both men and women (Carr, 2001).

Although all participants discussed increasing individualism, loss of identity, migration, and increasing socio-economic inequalities as factors that undermine community life, not everyone applied concerns directly to their community. We believe that this is partly related to the presence of a committed local partnership at one of our study locations which organised and implemented intergenerational projects aiming to encourage community involvement, as well as projects related to regeneration and renewal such as the continuing improvement of the local park environment. Fan et al. (2011), for example, emphasised the positive interrelation between park spaces, PA, stress mitigation and social support. They highlighted that the opportunity to socialise in public green spaces strengthens their health benefits if they are accessible and multifunctional. Furthermore, the urban area we used for two of our focus groups had undergone a considerable structural change as the closure of several collieries dissolved former communities with particular pit identities, and subsequent town expansion created relatively anonymous neighbourhoods.

Finally, our findings illustrate how gardening has remained a popular activity which is directly related to the industrial past of the areas (Bulmer, 1978; Zweig, 1948). For many pit families, allotments provided an essential food supply and an opportunity to experience social recognition. This is different from other findings where most of the participants who were very active in their jobs did not engage in physically demanding leisure-time activities because they were either too tired or had other priorities (Wandel and Roos, 2005). Today, however, limited access to and availability of allotments, particularly in more urban areas, as well as a tendency to more sedentary lifestyles has influenced former activity patterns, and the original purpose of gardening has shifted from being a food-producing and socio-cultural necessity towards a more exclusive leisure-time activity.

There are limitations to our study. This research was dependent on the co-operation of the working men's clubs and their members, and it was challenging to recruit an appropriate study sample. Similar to other research projects applying focus group methodology (Hoebeke, 2008), this study is therefore limited in size. Initially, women were hesitant to participate which may partly reflect the intrinsic male-dominated environment of the clubs. As our study focussed on change related to the communities' socio-economic history, the participants were relatively homogeneous in age. Although some participants attributed components of their physical decline to employment in mining it was not possible to assess this further as we had no comparator group of individuals from non-mining areas. Therefore some changes observed may have been more generally associated with declines in age-related physical function. Further, the attendees were highly motivated, very active community members and partly experienced in fundraising and communicating community issues to stakeholders. The transferability of our results has therefore to be considered in the context that they reflect attitudes and perceptions of a particular group in a specific socio-cultural setting.

Prior research has highlighted the particular challenge to reach and motivate residents of low-income communities to participate in health enhancing programmes and activities (Withall et al., 2010; Yancey et al., 2006). We therefore believe that our findings have implications for policy and practice. The important role of community organisation for health education and practice has previously been highlighted (Minkler et al., 2008). As the historically hard working conditions in ex-mining communities have limited particularly older residents' physical abilities, the development and availability of programmes that adequately consider age, health status and accessibility could facilitate the participation of those who may believe that their personal circumstances and living conditions exclude them from pursuing a sufficiently active lifestyle. There are some promising examples for health enhancing community programmes for senior residents such as the implementation of healthy living networks which support and encourage residents to take and keep control over their independence and well-being (Henderson, 2011; Thomson, 2010). Time banking, for example, is a reciprocal skill and service exchange which encourages neighbourly help and builds trust within communities through the generation of social capital and the reduction of social exclusion. The availability of these programmes is, however, limited and their continuity frequently uncertain.

We suggest it could be beneficial to focus further investigations on intergenerational factors that impact PA behaviours across different age-groups in deprived neighbourhoods. We noted that there was a strong and consistent view expressed by our study participants that the social functioning of neighbourhoods was limited by a gulf in understanding between the young versus the long-term inhabitants. Such initiatives could help to develop interventions where political as well as family and community structures complement and consolidate each other. For example, the reinforcement of family and community support could mitigate factors related to changing and challenging living conditions. Our participants emphasised that local projects such as the re-development of public green spaces as well as recreational country parks representing industrial history have recently gained an increase in users and visitors (Joint Committee of Beamish Museum (2011)), and similar success has been reported from other areas facing post-industrial structural change (Goch, 2002). The emotional link to the socio-cultural history of areas has been shown to be a strong motivator for communities to tackle adverse effects of industrial decline and evaluate future needs (Mellor and Stephenson, 2005). We believe that this potential is a relatively unexplored but invaluable source to enhance PA in areas characterised by persisting post-industrial decline.

## Figures and Tables

**Table 1:** Characteristics of the study sample.

	Group 1	Group 2	Group 3	Group 4
Number of participants	4	6	4	5
Gender	Female	Male	Male	Female
Average age	70	73	77	67
Working status				
Retired	4	4	4	5
Not in paid employment	0	2	0	0
Number of participants with a working history directly related to the mining industry	0	6	3	0
Self-reported health status				
Good	2	0	0	2
Fair	1	3	4	3
Bad	1	3	0	0
Average years of residence in the current community (at least 30 years)	59	66	59	48
Community classification	Town	Town	Village	Village
