# Supplementary material for: “I used to be as fit as a linnet” – Beliefs, attitudes, and environmental supportiveness for physical activity in former mining areas in the North-East of England
Source: Soc Sci Med. 2015 Feb;126:110–8. doi: 10.1016/j.socscimed.2014.12.002 (PMC4310854; doi:10.1016/j.socscimed.2014.12.002)
Supplement: Supplementary file 1 [file mmc1.docx]

# Additional material A

**Questionnaire**

Would you please fill in this short questionnaire? It will provide some background information about the characteristics of the people who are here today. All of your answers will remain anonymous in any reports.

Date___________________________

Time___________________________

Name

**Are you**

- male or
- female?

**What is your ethnic background?**

- White
- Mixed
- Asian or Asian British
- Black or Black British
- Chinese
- Other___________________________________

**What is your current work status?**

- In full-time paid employment
- In part-time paid employment
- Not in paid employment
- Looking after home/family
- Retired
- Permanently sick
- Other__________________________________

**How would you describe your health?**

- good
- fair
- bad

**Have you ever worked in the mining industry?**

- No
- Yes
- If yes, what was your job? ____________________________________

**Which community do you live in? ____________________________________**

**For how many years have you lived in this community? __________________**

**What is your year of birth?** __________
