# Supplementary material for: “I used to be as fit as a linnet” – Beliefs, attitudes, and environmental supportiveness for physical activity in former mining areas in the North-East of England
Source: Soc Sci Med. 2015 Feb;126:110–8. doi: 10.1016/j.socscimed.2014.12.002 (PMC4310854; doi:10.1016/j.socscimed.2014.12.002)
Supplement: Supplementary file 2 [file mmc2.docx]

# Additional material B

**Questioning route**

**Opening**

Introduction of participants to research project, exchange of names

**Introductory theme**

**Q1** Do people know each other in your neighbourhood?

**Q2** In what sort of things do you get involved in your community?

**Q3** Are there things you particularly like or dislike about your community as a place to live?

**Transition theme**

**Q4** In some places people have worse health than in others. Can you think of any reason for that?

**Q5** Which of those are particularly important in your community?

**Q6** If you think about your community, would you say that the health of the residents has changed over the last 30 years?

**Q7** What factors would you say have led to those changes?

**Q8** What about the closure of the collieries in this area? Would you say that has anything to do with health in your community?

**Key questions theme**

[Choosing of newspaper headlines; “+ positive” or “- negative” connotation.]

**Q9** Please let us know why you chose this headline.

- District Council helps communities to live healthier and more fulfilling lives
- District Council helps residents to take greater personal responsibility for their health
- Join a fun packed programme of fitness and healthy eating sessions
- Gardening for health – nature’s own fitness centre
- Be cool, walk to school
- Golf your way to a longer life
- Good fitness offers hope for fatigue and depression
- Get a dog and walk for health
- It is never too late to start
- Regular walking slashes risk of stroke dramatically
- Poor people are less healthy than rich people
- Cyclist run over by car
- Running is bad for your knees
- Local park needs makeover
- Potholes everywhere
- Greatest health risk isn’t cancer or heart disease - it’s lack of exercise
- Eating, drinking and watching TV cause more deaths than smoking
- High crime rates upset local residents
- Smoking and drinking for comfort
- Drug use increased dramatically over the last years

**Q10** People in some places do more walking, gardening, or other things that doctors say are good for health than people from other places. Do you have any ideas what the reasons for that may be?

**Q11** How important are activities like walking and gardening in your life?

**Ending questions**

**Q12** If you were in a discussion with local politicians or health experts: what would be the most important thing that you would suggest to them that needs to change to improve health in your community?

**Q13** I needed your help to learn more about health in your community and where things are particularly good or bad. I wanted to know what helps you to do something for your health and what your council and politics in general could do to support your needs better. Is there anything that we missed? Is there anything that you would like to add?
